# Spectroscopic and molecular docking studies on binding interactions of camptothecin drugs with bovine serum albumin

**DOI:** 10.1038/s41598-025-92607-3

**Published:** 2025-03-07

**Authors:** Yuhe Wang, Junfeng Li, Xuanda Li, Bingmiao Gao, Jiao Chen, Yun Song

**Affiliations:** Engineering Research Center of Tropical Medicine Innovation and Transformation of Ministry of Education & International Joint Research Center of Human-Machine Intelligent Collaborative for Tumor Precision Diagnosis and Treatment of Hainan Province & Hainan Provincial Key Laboratory of Research and Development on Tropical Herbs, School of Pharmacy, Hainan Medical University, Haikou, 571199 Hainan People’s Republic of China

**Keywords:** Bovine serum albumin, Camptothecin drugs, Binding interactions, Fluorescence Titration, Molecular Docking, Medical research, Molecular medicine

## Abstract

This study investigates the binding interactions between bovine serum albumin (BSA) and camptothecin (CPT) drugs (camptothecin, 10-hydroxycamptothecin, topotecan, and irinotecan) using UV–Vis spectroscopy, fluorescence spectroscopy, three-dimensional fluorescence spectroscopy, and molecular docking techniques. The fluorescence quenching of BSA by CPT drugs follows a static mechanism, with binding constants (K_b_) ranging from 4.23 × 10^3^ M^− 1^ (CPT) to 101.30 × 10^3^ M^− 1^ (irinotecan), demonstrating significant drug binding selectivity. Thermodynamic analysis reveals distinct interaction mechanisms: topotecan binding is driven by hydrogen bonding (ΔH = − 10.96 kJ·mol^− 1^) and hydrophobic interactions (ΔS = 0.066 kJ·mol^− 1^·K^− 1^), while irinotecan exhibits stronger binding dominated by electrostatic forces (ΔH = − 86.77 kJ·mol^− 1^) with significant entropy loss (ΔS = − 0.161 kJ·mol^− 1^·K^− 1^). Molecular docking confirms preferential binding at Sudlow site I of BSA, with hydrophobic interactions and hydrogen bonding as the primary driving forces. These findings provide a comprehensive understanding of CPT-BSA interactions, offering valuable insights for the design of albumin-based drug delivery systems with optimized pharmacokinetic profiles.

**Supplementary Information:**

The online version contains supplementary material available at 10.1038/s41598-025-92607-3.

## Introduction

**Fig. 1:**
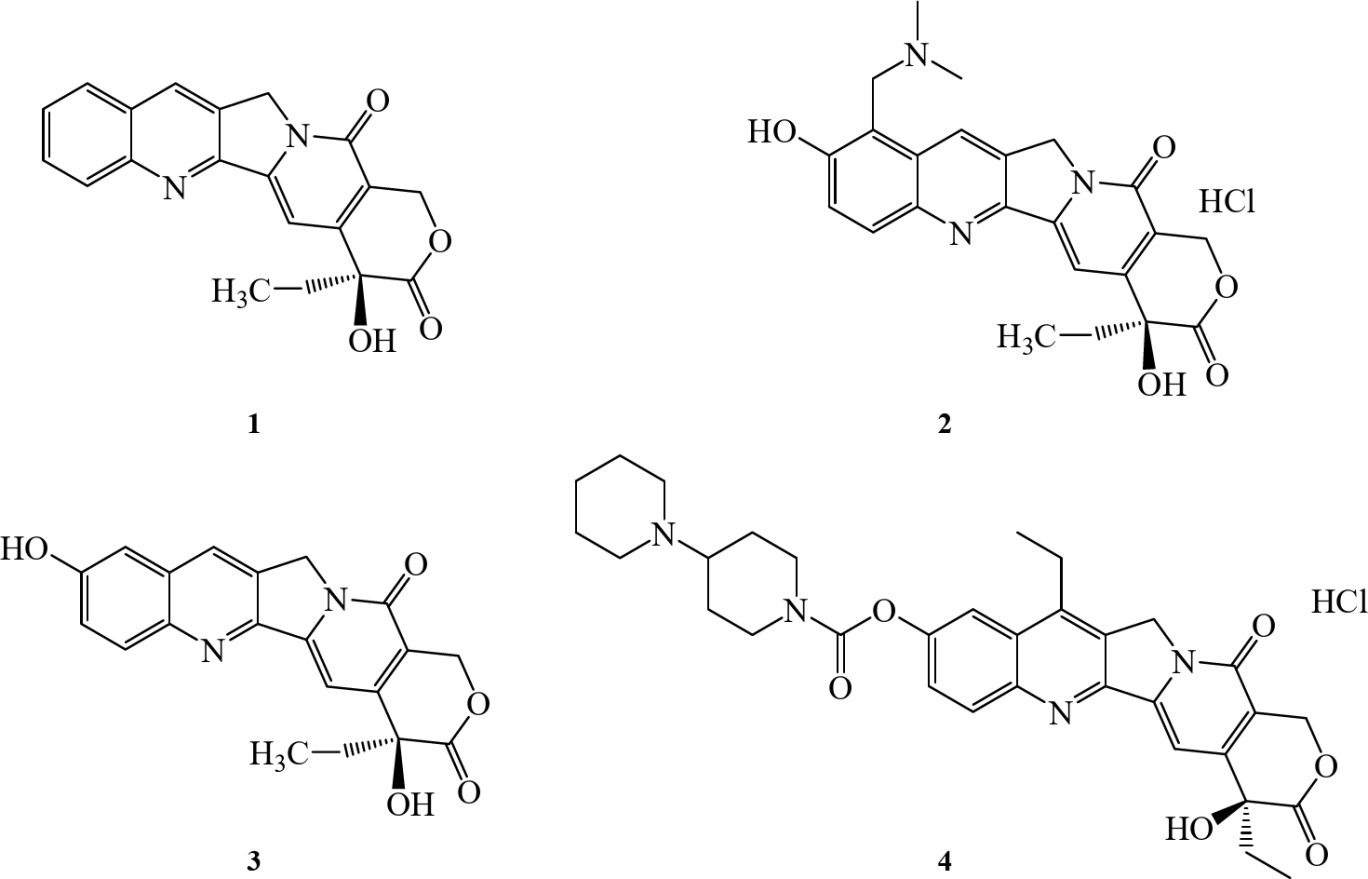
Chemical structure of CPT drugs, Camptothecin (CPT) (1), Topotecan (TPT) (2), 10-Hydroxycamptothecin (10-HCPT) (3), and Irinotecan (CPT-11).

Camptothecin (CPT) is a pentacyclic quinoline alkaloid extracted from the roots, bark, and fruits of Camptotheca acuminata^1^. It specifically targets DNA topoisomerase I (TopI), forming a stable “drug-TopI-DNA” ternary complex, which disrupts DNA replication and exhibits potent antitumor activity^2,3^. As a natural topoisomerase inhibitor, CPT is effective against various cancers, including lung, gastric, bladder, and breast cancers, highlighting its broad potential in cancer therapy^4^. Despite its unique antitumor mechanism, CPT’s clinical application is severely limited by its extremely low water solubility, poor stability, short half-life, and significant toxic side effects^5,6^. To tackle these challenges, researchers have focused on structural modifications of CPT, resulting in the development of numerous derivatives. Some of which exhibit enhanced water solubility and stability while maintaining potent antitumor efficacy^7–9^. CPT and its derivatives have shown significant anticancer effects, with several being approved for clinical use. Their structures are illustrated in Fig. 1. Topotecan (TPT), a water-soluble derivative, is widely used for treating ovarian and small cell lung cancers by inhibiting topoisomerase I^10^. 10-Hydroxycamptothecin (10-HCPT) effectively treats tumors such as gastric, lung, and colorectal cancers, inhibiting tumor cell proliferation and inducing apoptosis^11^. Irinotecan (CPT-11), particularly effective for colorectal cancer, must be metabolized to its active form, SN-38, to exert its topoisomerase I inhibitory effects^12,13^. Despite their success, CPT drugs face significant challenges. Both CPT and 10-HCPT suffer from poor water solubility, which limits their bioavailability^14^. Additionally, TPT and CPT-11 can cause severe side effects such as myelosuppression and gastrointestinal reactions, restricting their dosage and efficacy^15,16^. These drugs are also prone to developing resistance, thereby reducing their therapeutic effectiveness^17^.

Encapsulating CPT drugs in nanocarriers is essential for reducing the toxic side effects of antitumor drugs and improving their bioavailability and solubility^18^. Although various formulations, such as liposomes^19,20^, polymeric micelles^21–23^, and nanoparticles^24,25^, have been developed, challenges persist due to the characteristics of the carrier materials. Conventional formulations, including liposomes, polymeric micelles, and solid lipid nanoparticles, often encounter issues such as poor in vivo stability, low drug loading capacity, and burst release.

Serum albumin (SA) is the most abundant protein in blood, constituting 55–65% of total plasma proteins. It plays critical roles such as maintaining plasma colloid osmotic pressure and transporting substances like fatty acids, hormones, and drugs^26^. Bovine serum albumin (BSA) and human serum albumin (HSA) are particularly well-suited as drug carriers due to their biocompatibility, extended circulation time, targeting ability, modifiability, and solubilization properties^27–29^. Current SA-based drug delivery systems, including albumin nanoparticles and albumin-bound drugs, show promising potential in fields such as anticancer, anti-inflammatory, and protein drugs^30^. Abraxane^®^, the first HSA-based anticancer drug approved by the US FDA, is used clinically to treat metastatic breast cancer, non-small cell lung cancer, and pancreatic cancer^31^. By employing HSA as a carrier, Abraxane^®^ enhances the solubility and bioavailability of paclitaxel, significantly improving its safety profile and ease of administration, thereby providing patients with a superior treatment option.

Investigating the binding interactions between CPT drugs and BSA is crucial for guiding the formulation of drug-loaded nanoparticles. Understanding these interactions can optimize the drug-loading capacity, release rate, and targeting ability of nanoparticles, thereby enhancing drug delivery efficiency and specificity. This knowledge also informs adjustments to the surface properties and functional modifications of nanoparticles to ensure optimal pharmacokinetic and pharmacodynamic characteristics in vivo. Establishing the binding mechanism, binding constants, and binding sites of CPT drugs with BSA provides a solid foundation for developing effective and safe drug-loaded nanoparticles.

This study utilized UV spectroscopy, fluorescence spectroscopy^32^, and molecular docking simulations^33^ to comprehensively investigate the interaction between BSA and CPT drugs. Fluorescence spectral analysis provided quantitative binding constant information, allowing precise assessment of the binding strength between the drugs and albumin. Concurrently, molecular docking simulations visualized the three-dimensional structure of drug-protein complexes at the atomic level, revealing key binding sites and interaction forces. This multi-method approach not only deepens understanding of the encapsulation mechanism of CPT drugs with BSA but also establishes a crucial theoretical foundation for developing new albumin carrier systems and optimizing drug delivery strategies^34,35^.

## Results and discussion

### UV spectroscopy

UV absorption measurement is a simple yet effective technique for examining structural changes and complex formation^36^. The absorption spectrum of BSA typically displays two characteristic peaks, around 210 nm and 280 nm. Variations in the absorbance near 280 nm signify interactions with small molecule drugs. A slight red shift indicates increased polarity around aromatic amino acid residues, while a blue shift suggests decreased polarity or increased hydrophobicity around these residues^37^.

**Fig. 2:**
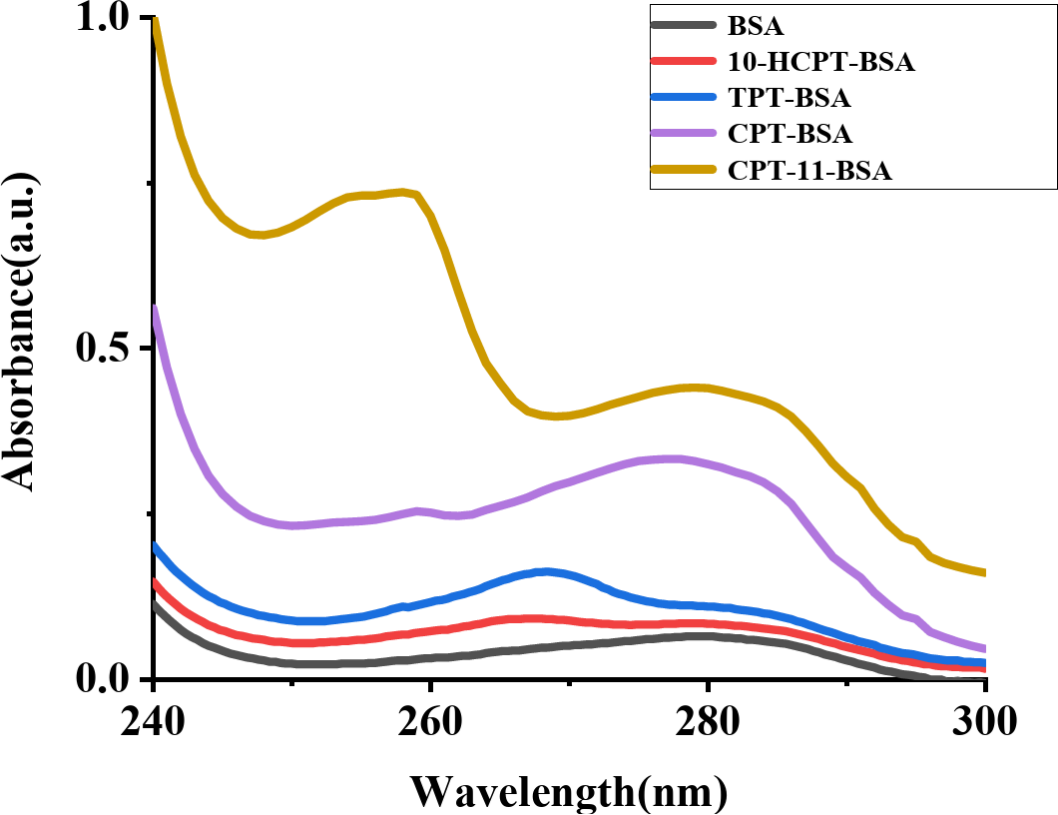
UV absorption spectra of BSA (1.0 × 10^− 6^ mol/L) in aqueous phosphate buffer solution (298.15 K), both in the absence and presence of CPT drugs (CPT, 10-HCPT, TPT, and CPT-11) at a fixed concentration of 5.0 × 10^− 6^ mol/L.

**Table 1:** Changes in UV absorption peaks of BSA upon binding of CPT drugs.

Drugs	Changes in absorption peaks (280 nm)
CPT	280 nm→277 nm
TPT	280 nm→268 nm
10-HCPT	280 nm→278 nm
CPT-11	280 nm→258 nm

This study evaluated the changes in the UV absorption spectrum of BSA (1.0 × 10^− 6^ mol/L) upon addition of CPT drugs, as shown in Fig. 2. Table 1 indicates that following the addition of CPT, TPT, 10-HCPT, and CPT-11, the UV absorption peak of BSA at 280 nm exhibited blue shifts of 3 nm, 12 nm, 2 nm, and 22 nm, respectively. These shifts arise from interactions between CPT drugs and BSA, which alter the electronic structure and energy levels of BSA molecules upon complex formation^38,39^.

The observed blue shift indicates increased hydrophobicity around aromatic amino acid residues, such as tryptophan, tyrosine, and phenylalanine. The varying extents of blue shifts are likely related to each drug’s specific binding sites, affinities, and interaction modes with BSA. The significant blue shifts induced by TPT and CPT-11 suggest more stable or extensive hydrophobic interactions with BSA, whereas the minor shifts with CPT and 10-HCPT indicate minimal changes in BSA’s environment, reflecting a slight reduction in polarity.

### Fluorescence spectroscopy analysis

Fluorescence spectroscopy is an effective technique for studying drug-BSA interactions^40^, primarily due to the intrinsic fluorescence of tryptophan (Trp) and tyrosine (Tyr) residues in BSA^41,42^. Spectral titrations of BSA with CPT drugs were conducted in an aqueous phosphate buffer (pH 7.40) to quantitatively evaluate the inclusion complexation behavior. The inner filter effect (IFE) arises when high analyte concentrations (e.g., fluorophores, ligands, or biomolecules) attenuate excitation light or absorb emitted photons, effectively reducing the optical path length and consequently causing depressed fluorescence readings. To mitigate this phenomenon in our experimental system, we strategically optimized sample concentrations through preliminary calibration studies^43^. So, the BSA concentration was held constant at 2.0 × 10^− 6^ mol/L, while CPT drug concentrations varied up to 16.5-fold. The observed spectral changes, indicative of a host-guest inclusion complex, resulted in fluorescence quenching or enhancement.

**Fig. 3:**
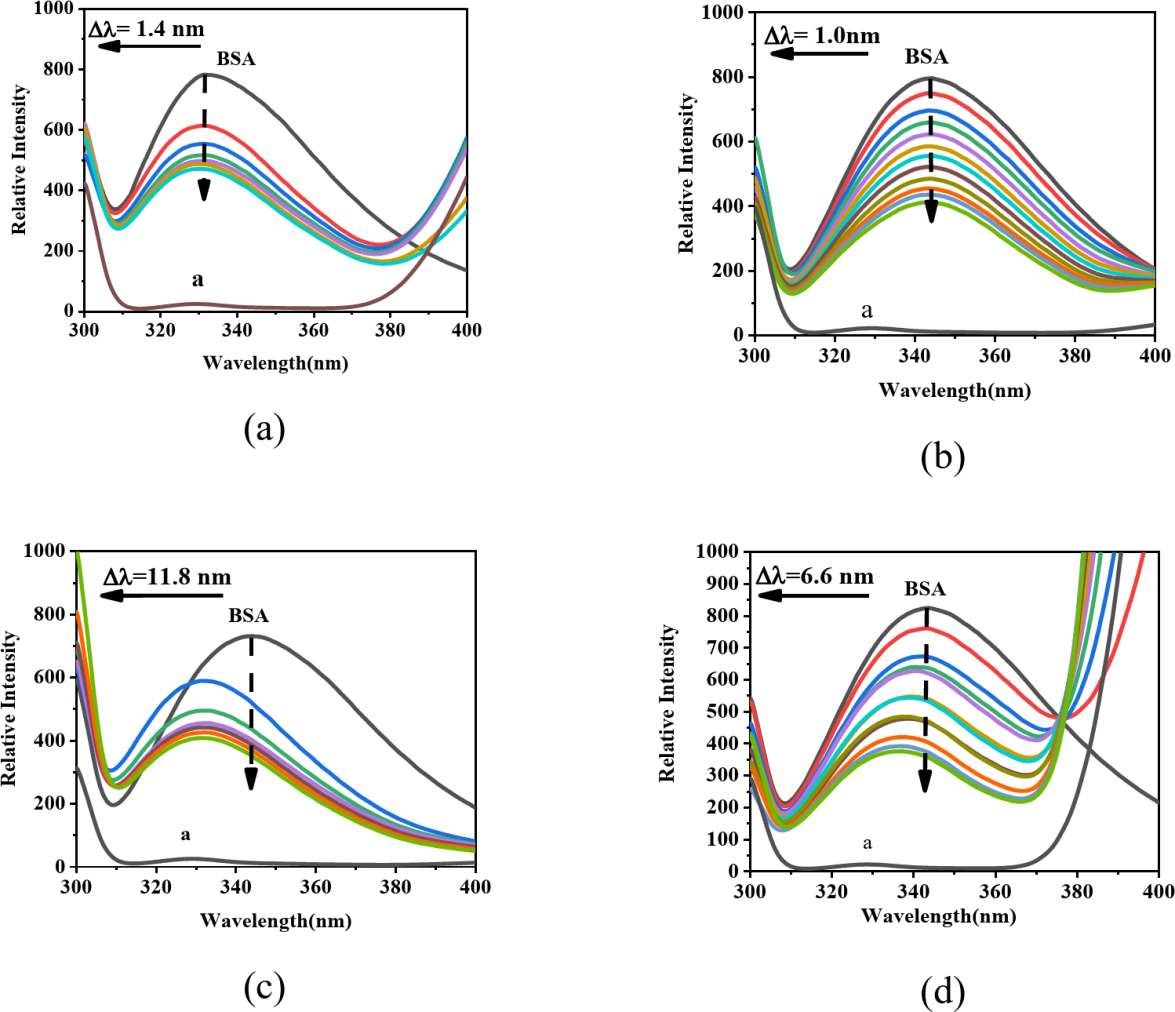
Changes in the fluorescence spectra of BSA (2 × 10^− 6^ mol/L) upon the addition of CPT drugs, (**a**) CPT; (**b**) TPT; (**c**) 10-HCPT and (**d**) CPT-11 in aqueous phosphate buffer solution.

As depicted in Fig. 3, the fluorescence intensity of BSA decreased with the addition of each CPT drug, confirming the formation of inclusion complexes within the BSA cavities^44^.

Fluorescence spectroscopy results showed a notable blue shift in the fluorescence emission wavelength (Δλ_max_) of BSA as CPT drug concentrations increased. Figure 3 illustrates that the maximum fluorescence emission peak of BSA shifted by 1.4 nm, 1.0 nm, 11.8 nm, and 6.6 nm with the addition of CPT, TPT, 10-HCPT, and CPT-11, respectively. These variations indicate different interactions between each CPT drugs and BSA. The blue shift suggests reduced environmental polarity around amino acid residues, implying potential conformational changes in BSA upon drug binding, affecting drug affinity, binding sites, and spatial arrangement. Notably, 10-HCPT caused a significant 11.8 nm shift, indicating a substantial impact on BSA conformation.

Changes in frequency-dependent fluorescence intensity suggest that CPT drug binding to BSA is a multi-step process, involving initial hydrophobic interactions followed by stronger hydrogen bonding or electrostatic interactions. The blue shift in fluorescence, along with intensity changes, reveals the complex interaction mechanisms between CPT drugs and BSA^45,46^.

### Stoichiometry of CPT drugs-BSA complexes

**Fig. 4:**
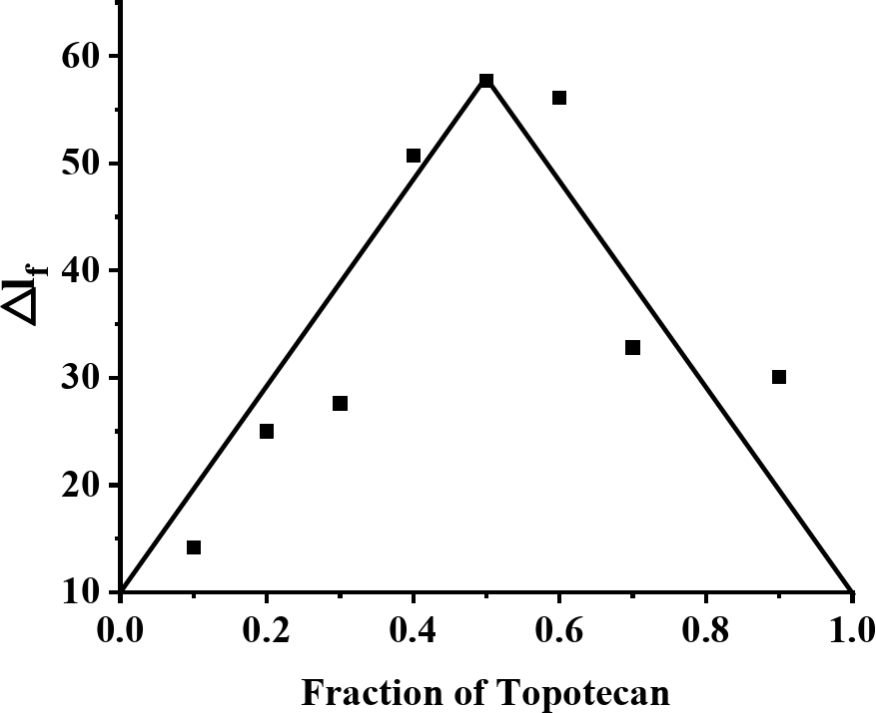
Job’s plot for the complexation of BSA with TPT in aqueous phosphate buffer solution ([BSA] + [TPT] = 2.0 × 10^− 6^ mol/L) produced with data taken from fluorescence spectra (λ_ex_ = 295 nm).

The stoichiometry of the inclusion complexation between BSA and a representative CPT drugs (TPT) was determined using Job’s method. In this experiment, the total concentration of TPT and BSA was kept constant at ([BSA] + [TPT] = 2.0 × 10^− 6^ mol/L), while their molar fractions were varied. This allowed us to plot the change in fluorescence intensity (ΔF) against the molar fraction of TPT, as shown in Fig. 4. The fluorescence intensity reached a maximum at a TPT molar fraction of 0.5, indicating a 1:1 inclusion complexation. This suggests that CPT drugs interact with a single binding site in BSA, with an n value of 1. The 1:1 binding ratio provides critical insight into the interaction between CPT drugs and BSA, indicating that BSA can bind CPT drugs in an equimolar fashion.

### Quenching mechanism

Fluorescence quenching mechanisms can be categorized into static, dynamic, and combined quenching^47^. Static quenching occurs when the fluorophore (BSA) and quencher (CPT drugs) form a ground-state complex, while dynamic quenching arises from collisional interactions. Combined quenching involves both mechanisms, where the fluorophore collides with and forms a stable complex with the quencher^48^. These processes can be described by the Stern-Volmer equation^49^:

$$\:{F}_{0}/F=1+{K}_{q}{\tau\:}_{0}\left[Q\right]=1+{K}_{sv}\left[Q\right]$$

**Fig. 5:**
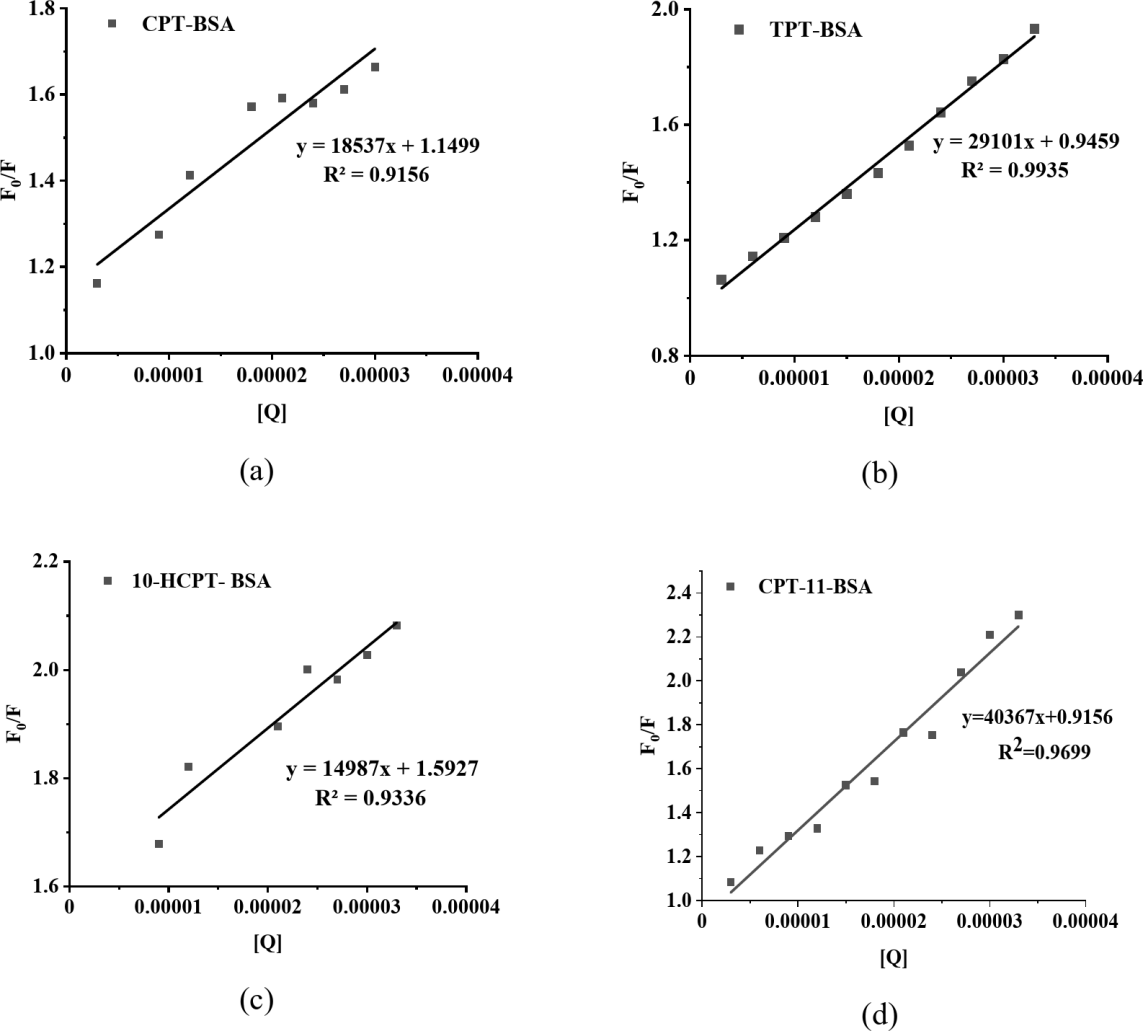
Stern-Volmer plot depicting the interaction between (**a**) CPT, (**b**) TPT, (**c**) 10-HCPT, and (**d**) CPT-11 and BSA.

Where F_0_ and F are the fluorescence intensities in the absence and presence of the quencher, respectively; K_SV_ is the Stern-Volmer quenching constant; [Q] is the quencher concentration; K_q_ is the bimolecular quenching constant; and τ_0_ is the fluorescence lifetime in the absence of the quencher (6.3 × 10^− 9^ s)^50^. By measuring the changes in BSA fluorescence intensity with varying concentrations of CPT drugs, we plotted a Stern-Volmer graph of F_0_/F against the concentration of CPT drugs [Q], as shown in Fig. 5. The X-axis represents the concentration of CPT drugs [Q] (unit: mol/L), and the Y-axis represents the fluorescence intensity ratio F_0_/F. The data points represent the experimentally measured F_0_/F values, while the solid line is the linear fit. Within the experimental concentration range, F_0_/F shows a strong linear relationship with [Q], consistent with a static quenching mechanism. The slope obtained from the linear fit according to the Stern-Volmer equation represents the quenching constant (K_SV_). The fitting data are summarized in Table 2. The corresponding correlation coefficients (R²) of the linear fits are all greater than 0.91, indicating excellent linear fitting.

**Table 2:** Stern–Volmer analysis data of the interaction between CPT drugs and BSA.

Ligand	Stern–Volmer equation			K_sv_ (×10^3^ L·mol^− 1^)	K_q_ (×10^11^ L·mol^− 1^·s^− 1^)
	Intercept	Slope	*R* ^2^		
CPT	1.15 ± 0.05	18536.73 ± 2298.23	0.91556	18.53	29.41
TPT	0.95 ± 0.02	29101.32 ± 782.38	0.99354	29.10	46.19
10-HCPT	1.59 ± 0.04	14986.88 ± 1787.60	0.93359	14.99	23.79
CPT-11	0.92 ± 0.05	40367.14 ± 2371.32	0.96988	40.37	64.08

CPT’s core structure is a five-ring system with a lactone ring, characterized by rigidity and hydrophobicity. Its interaction with BSA primarily involves hydrophobic interactions and van der Waals forces. Due to CPT’s simple structure and low polarity, changes in binding sites are minimal, leading to a lower K_SV_ value of 18.53 × 10³ M^− 1^. TPT resembles CPT but includes an additional N-methyl group, increasing its polarity and potential for hydrogen bonding. This increased polarity enhances interactions with BSA’s polar residues, resulting in a K_SV_ of 29.10 × 10^2^ M^− 1^. 10-HCPT, a CPT derivative with a hydroxyl group at the 10th position, also exhibits increased polarity and hydrophilicity, thereby slightly raising its K_SV_ to 19.56 × 10³ M^− 1^. CPT-11 has a significantly more complex structure, featuring a large hydrophilic phenylpiperazine group, which increases its polarity and strengthens hydrogen bonding and electrostatic interactions with BSA, leading to the highest K_SV_ of 40.37 × 10^2^ M^− 1^.

Incorporating hydrophilic groups into these drugs, such as the N-methyl group in TPT, the hydroxyl group in 10-HCPT, and the phenylpiperazine group in CPT-11, enhances their interactions with BSA, thereby increasing the quenching constant. Factors including molecular polarity, steric hindrance, hydrogen bonding, and hydrophobic interactions collectively determine the binding strength of these drugs with BSA, resulting in variations in K_SV_.

The bimolecular quenching rate constant (K_q_) is calculated using the formula K_q_ = K_SV_/τ_0_. The all calculated K_q_ values exceed 2.0 × 10^10^ L·mol^− 1^·s^− 1^, which is significantly higher than the maximum possible value for diffusion-controlled quenching (~ 1.0 × 10^10^ L·mol^− 1^·s^− 1^), further supporting a static quenching mechanism between CPT drugs and BSA^51^.

In general, the fluorescence lifetime measurement is the most definitive method to distinguish between dynamic and static quenching^52^. Time-resolved fluorescence experiments for both free BSA and binary (CPT drugs-BSA) systems were performed as illustrated in Fig. S1 and Table 3.

**Table 3:** Time-resolved fluorescence decay parameters for BSA and CPT drugs-BSA systems.

	A_1_	τ_1_ (ns)	A_2_	τ_2_ (ns)	*R* ^2^	τ_ave_ (ns)
BSA	0.84608	2.15289	0.16702	6.39407	0.9977	3.72
CPT-BSA	0.78549	1.88892	0.22632	5.77982	0.99732	3.71
10-HCPT-BSA	0.88064	2.23869	0.18582	7.483	0.99597	4.41
TPT-BSA	0.86363	1.69856	0.15431	7.6602	0.99739	4.36
CPT-11-BSA	0.76501	2.44608	0.29087	7.79033	0.99732	5.37

Free BSA shows an average fluorescence lifetime τ_ave_ of 3.72 ns, consistent with its intrinsic tryptophan emission. Binding with CPT minimally alters τ_ave_ 3.71 ns, suggesting weak environmental perturbation. In contrast, CPT analogs (10-HCPT, TPT, CPT-11) significantly increase τ_ave_ to 4.41 ns, 4.36 ns, and 5.37 ns, respectively. This indicates enhanced shielding of tryptophan residues, reducing non-radiative decay. CPT-11-BSA exhibits the longest τ_ave_, implying the strongest interaction or rigid microenvironment. Increased amplitude (A_2_) for analogs (e.g., CPT-11: 0.291) highlights a greater proportion of fluorophores in protected regions. High (R^2^) values (> 0.995) confirm reliable fitting. The binding of CPT to BSA primarily occurs through a static quenching mechanism.

### Binding constants between BSA and CPT drugs

This study employs fluorescence titration to determine the binding constant between BSA and CPT drugs, thereby quantifying their interaction strength. The binding constant (K_b_) is calculated using the following formula:

$$\log ({{\text{F}}_0} - {\text{F}})/{\text{F}}=\log {{\text{K}}_{\text{b}}}+{\text{n}}\log [Q]$$

**Fig. 6:**
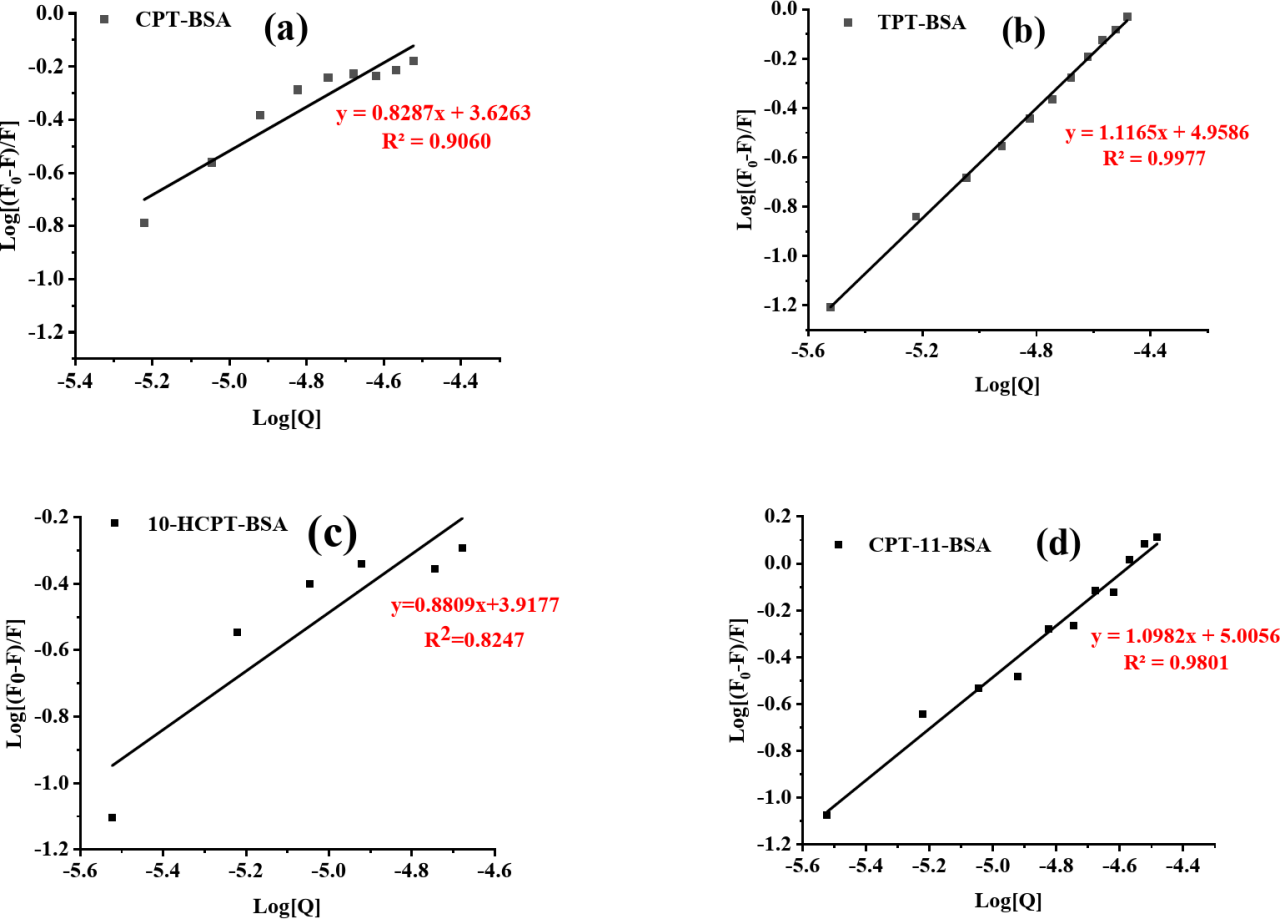
Double logarithmic plot of log(F_0_-F)/F against log[Q] for the fluorescence quenching data shown in Fig. 3.

Where K_b_ is the binding constant, n is the number of binding sites per BSA molecule, F_0_ and F are the fluorescence intensities without and with the quencher, respectively, and [Q] is the quencher concentration. A plot of log[Q] versus log[(F_0_−F)/F] is shown in Fig. 6. Linear regression is used to fit the data, resulting in a straight line whose slope indicates n (the number of binding sites), and the intercept gives logK_b_.

The free energy change ΔG for the interaction between CPT drugs and BSA was calculated using the equation:

$$\Delta {\text{G}}= - {\text{RT}}\ln {{\text{K}}_{\text{b}}}$$

Where R is 8.314 J·mol^-1^·K^-1^, T is 298 K (273 + 25 °C), and K_b_ is the calculated binding constant. The fitting data, along with the calculated K_b_ and ΔG, are presented in Table 4. With the number of binding sites close to 1, it implies that, on average, each BSA molecule binds to approximately one CPT drug molecule. This result aligns with the 1:1 stoichiometry determined using Job’s method. The binding stoichiometry analysis reveals distinct interaction patterns between BSA and CPT drugs. For CPT (*n* = 0.8287), approximately 0.83 molecules bind per BSA, indicating multiple binding sites with different affinities. Conversely, TPT (*n* = 1.1165) shows slightly more than one molecule binding per BSA, suggesting potential secondary binding sites or cooperative interactions. These differences reflect variations in molecular structures and binding affinities between the compounds.

**Table 4:** Binding constants (K_b_) and free energy changes (ΔG) of CPT drugs-BSA complexes.

Ligand	logK_b_ (L·mol^− 1^)	K_b_ (L·mol^− 1^)×10^3^	*n*	*R* ^2^	∆G (kJ·mol^− 1^)
CPT	3.6263	4.23	0.8287	0.9060	− 20.69
TPT	4.9586	90.91	1.1165	0.9977	− 28.29
10-HCPT	3.9177	8.27	0.8809	0.8247	− 22.35
CPT-11	5.0056	101.30	1.0982	0.9801	− 28.56

The binding constants (K_b_) for the interactions between BSA and CPT, TPT, 10-HCPT, and CPT-11 are 4.23, 90.91, 8.27, 101.30 × 10^3^ M^− 1^, respectively. CPT shows a relatively low K_b_ with BSA, suggesting weak affinity due to its simple five-ring structure with low polarity, resulting in predominantly hydrophobic interactions. TPT exhibits a significantly higher K_b_, indicating stronger affinity likely due to the N-methyl substituent, which enhances hydrogen bonding and electrostatic interactions. 10-HCPT has a K_b_ slightly higher than CPT’s, attributed to the added hydroxyl group which increases polarity and potential polar interactions, but not enough to significantly boost binding strength. CPT-11 has the highest K_b_, indicating very strong affinity, likely due to its complex structure with a large hydrophilic group, enhancing interactions through hydrogen bonding and electrostatic forces.

TPT and CPT-11 exhibit significantly higher K_b_ values with BSA, indicating stronger binding affinities that may lead to prolonged in vivo retention and increased bioavailability. In contrast, CPT and 10-HCPT have lower K_b_ values, suggesting weaker interactions with BSA primarily driven by hydrophobic forces, with limited contribution from polarity and steric effects. These results highlight the critical influence of molecular structure, especially the presence of polar groups and steric modifications, on binding strength to BSA. Additionally, the negative ΔG values for all CPT drugs indicate that the binding reactions are spontaneous.

The distinction between K_sv_ and K_b_ arises from their fundamental physical meanings and associated interaction mechanisms: K_sv_ (Stern–Volmer constant) reflects collisional quenching efficiency under dynamic quenching conditions. Dominated by short-range non-specific interactions (e.g., transient dipole–dipole effects). K_b_ (Binding constant) quantifies thermodynamic binding affinity in static quenching scenarios. Describes equilibrium for stable complex formation. Governed by specific non-covalent forces (e.g., hydrogen bonding, hydrophobic interactions, electrostatic forces)^53,54^.

The invariance of fluorescence lifetime and the calculated bimolecular quenching constants (K_q_) for all CPT drugs supporting a static quenching mechanism through complex formation.

This binding behavior demonstrates excellent physiological relevance, as the moderate K_b_ of TPT-BSA and CPT-11-BSA (10^4^–10^6^ M^− 1^) ensures optimal balance between plasma protein binding and drug bioavailability. The reversible nature of the interaction facilitates controlled drug release at target sites, while the involvement of multiple interaction forces (including hydrophobic interactions and hydrogen bonding) maintains complex stability without compromising BSA’s structural integrity or biological functions. These characteristics make the interactions beween TPT and CPT-11 with BSA particularly suitable for in vivo drug delivery applications, as it effectively balances drug transport and release kinetics to achieve optimal therapeutic efficacy.

### Interaction forces of TPT and CPT-11 between BSA

**Fig. 7:**
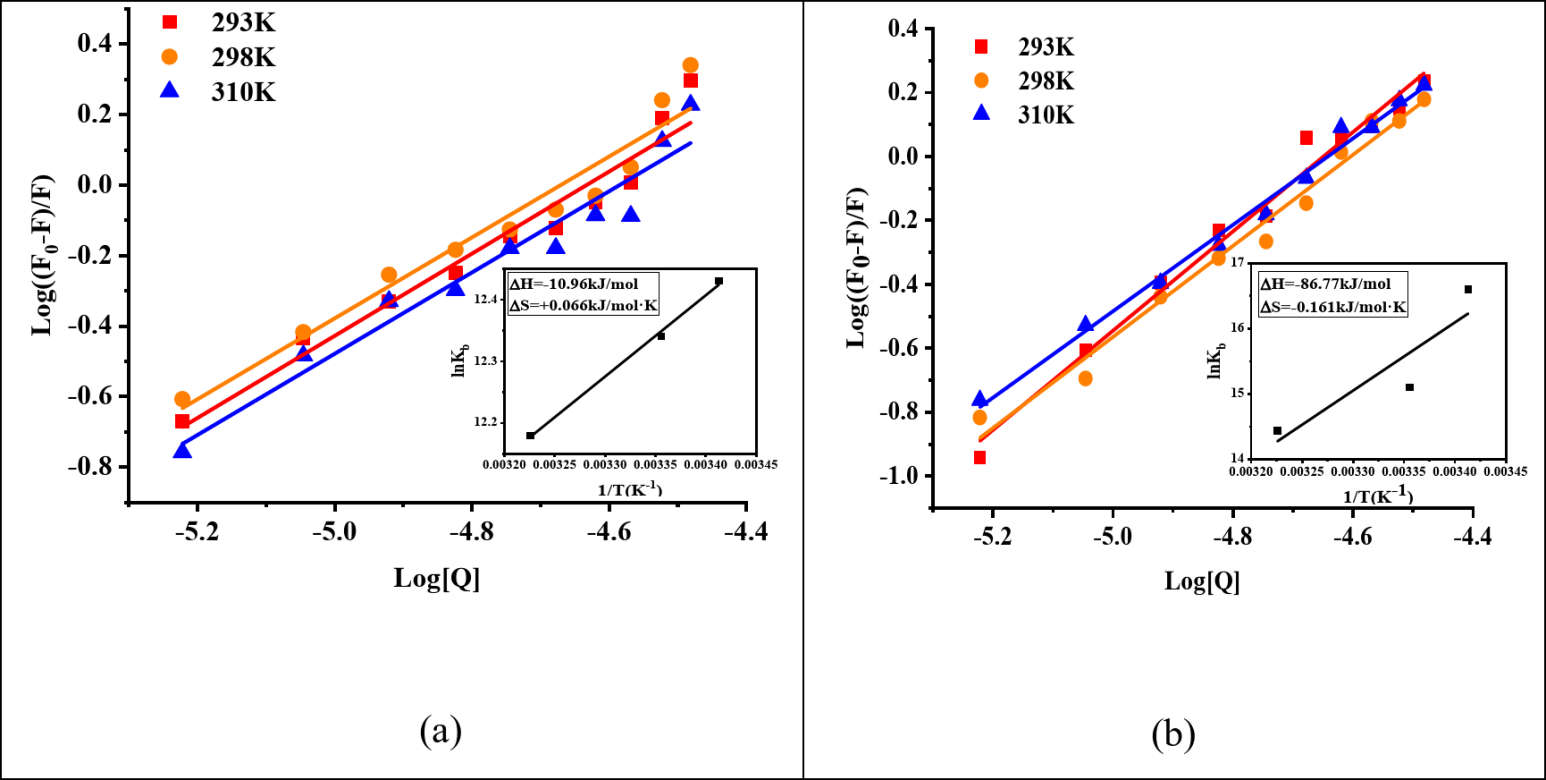
Double logarithmic plots of log(F_0_-F)/F versus log[Q] for the binding of (**a**) TPT and (**b**) CPT-11 to BSA, obtained at three different temperatures, i.e., 293, 298 and 310 K. Inset shows the van’t Hoff plot for the TPT-BSA and CPT-11-BSA systems.

As shown in Fig. 7; Table 5, thermodynamic profiling reveals distinct binding mechanisms for TPT and CPT-11 with BSA. For TPT, the enthalpy-entropy compensation (ΔH = -10.96 kJ·mol^− 1^) signifies hydrogen-bond-driven binding reinforced by hydrophobic effects, enabling temperature-modulated dissociation. In contrast, CPT-11’s strongly enthalpy-driven interaction (ΔH = − 86.77 kJ·mol^− 1^) with entropy penalty (ΔS = − 0.161 kJ·mol^− 1^·K^− 1^) suggests rigid complexation via multivalent electrostatic forces, which sharply weakens at elevated temperatures (K_b_) reduction > 88% at 310 K.

The physiological relevance of CPT drug-BSA binding is supported by the observed binding constants (K_b_ = 10^3^−10^5^ M^− 1^), which ensure effective plasma retention while allowing drug release at target sites. For instance, TPT’s moderate affinity (K_b_ = 251.19 × 10^3^ M^− 1^) balances prolonged circulation (t₁/₂ ≈ 8 h) with efficient dissociation in tumor microenvironments. Additionally, the temperature sensitivity of binding (e.g., CPT-11’s K_b_ decreases by 88% at 310 K) aligns with hyperthermia-triggered release strategies. These findings, combined with spontaneous binding thermodynamics (ΔG < 0), confirm the feasibility of albumin-mediated CPT delivery in vivo.

**Table 5:** Binding constants and thermodynamic parameters for TPT–BSA and CPT-11-BSA interactions, studied at three different temperatures, pH 7.4.

	T (K)	LogK_b_	K_b_ (×10^3^)	ΔG (kJ·mol^− 1^)	ΔH (kJ·mol^− 1^)	ΔS (kJ·mol^− 1^·K^− 1^)
TPT	293	5.40	251.19	− 30.30	− 10.96	+ 0.066
	298	5.36	229.09	− 30.63		
	310	5.29	194.98	− 31.42		
CPT-11	293	7.21	16218.10	− 39.60	− 86.77	− 0.161
	298	6.56	3630.78	− 38.79		
	310	6.27	1862.09	− 36.86		

### 3-D fluorescence spectra

**Fig. 8:**
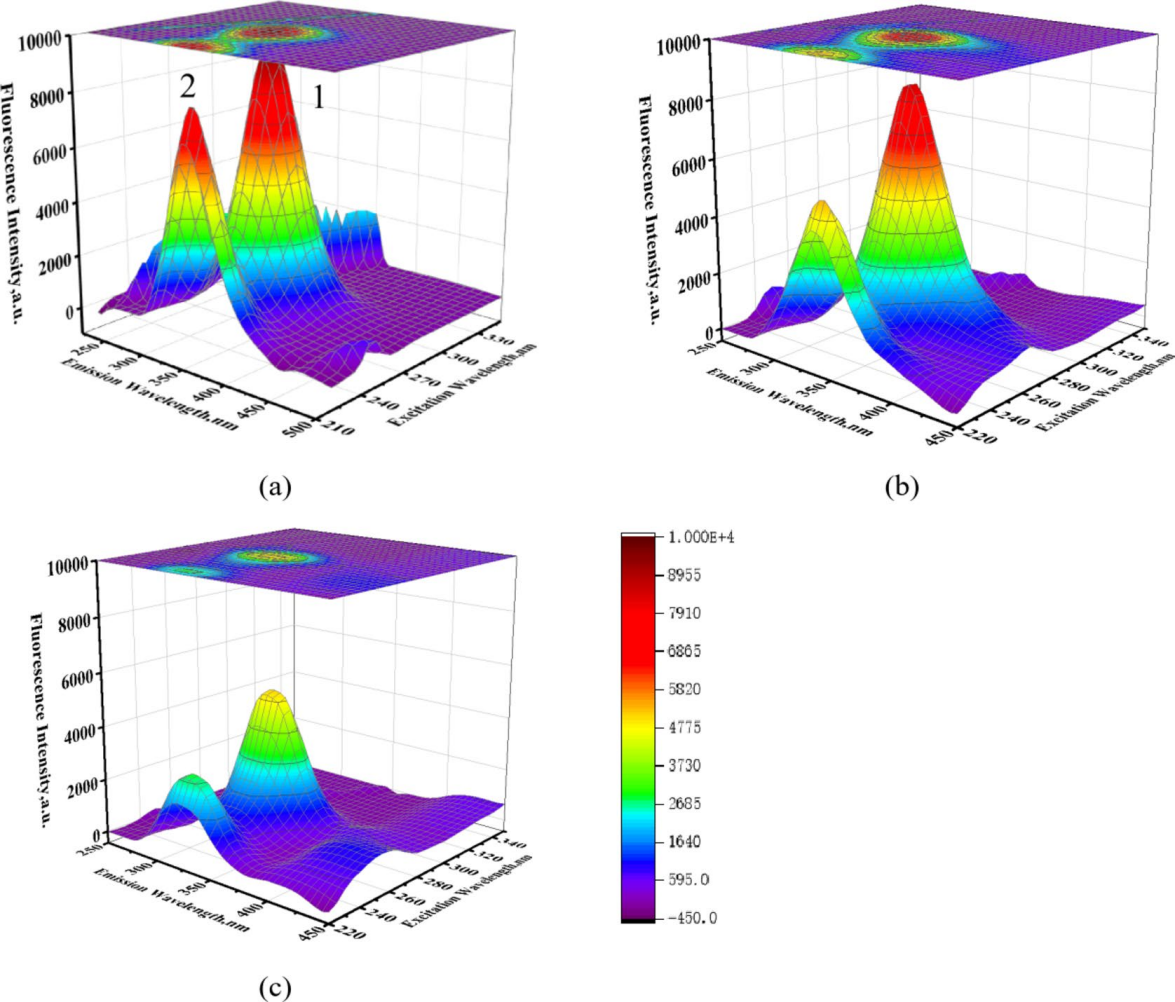
3-D fluorescence spectral projections and corresponding contour maps of (**a**) 3 × 10^−6^ mol/L BSA, (**b**) TPT-BSA (5:1) and (**c**) TPT-BSA (10:1) systems in aqueous phosphate buffer solution (pH 7.4).

Three-dimensional fluorescence spectroscopy, or excitation-emission matrix (EEM) spectroscopy, offers distinct advantages and broad applications for analyzing interactions between small molecules and BSA^55^. By examining changes in the 3-D fluorescence spectra of BSA in the presence of CPT drugs (Figure S2), we can discern microenvironmental perturbations around the Tyr and Trp residues induced by ligand binding. Figure 8 displays typical 3-D fluorescence spectra and contour maps for BSA (panel a) and TPT-BSA systems (panels b and c). Two prominent fluorescence peaks correspond to the Tyr and Trp residues in BSA. A comparative analysis of BSA’s 3-D fluorescence spectra with and without TPT (at a 10-fold molar excess) revealed intensity reductions of 49% and 65%, and blue shifts of 15 nm and 20 nm in the emission maxima of peaks 1 and 2, respectively.

**Fig. 9:**
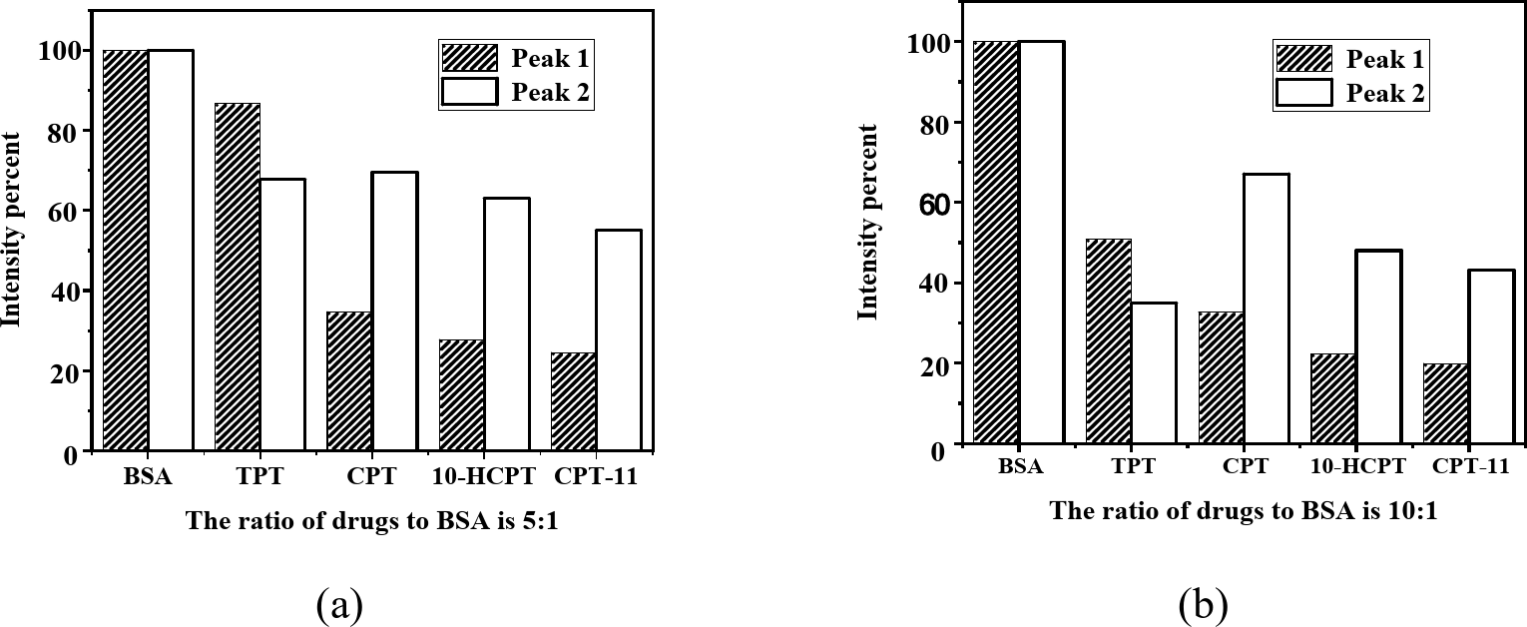
3-D fluorescence intensity variation chart (**a**) CPT drugs: BSA (5:1), (**b**) CPT drugs: BSA (10:1).

The decreases in fluorescence intensity at peaks 1 and 2 indicate CPT drugs-BSA interactions. Generally, greater fluorescence quenching correlates with higher binding affinity, with significant quenching suggesting stronger interactions. Changes at peak 1 typically reflect direct binding at specific BSA sites, while alterations at peak 2 are linked to modifications in BSA’s secondary structure or conformational changes induced by the drug. As shown in Fig. 9, among the drugs studied, CPT-11 shows the strongest binding affinity to BSA, evidenced by nearly complete quenching at peak 1, indicating strong interaction at the specific binding site. Additionally, significant quenching at peak 2 suggests considerable impact on BSA’s overall conformation, likely mediated by hydrogen bonding and electrostatic interactions.

Table S1 summarizes the 3-D fluorescence spectral analysis of CPT drug-BSA interactions. It includes data on peak excitation/emission wavelengths (λex/λem), fluorescence intensity, percentage reduction in intensity, and emission maximum shifts for different drug-to-BSA molar ratios (5:1 and 10:1). A blue shift in BSA’s maximum emission peak suggests that CPT drugs have entered BSA’s hydrophobic binding pocket, resulting in fluorescence emission at shorter wavelengths compared to the aqueous solution. The observed blue shift provides valuable insights into the interaction mechanisms between CPT drugs and BSA.

The chiral environment of BSA, due to its L-amino acid composition and α-helical structure, ensures stereoselective binding with CPT drugs, preserving the function of critical binding sites (e.g., Sudlow site I). This chirality-driven selectivity helps maintain BSA’s structural integrity while facilitating drug transport. The intermolecular forces, such as hydrophobic interactions and hydrogen bonding, between CPT drugs and BSA reduce conformational entropy but are energetically balanced by favorable enthalpy changes, stabilizing the complex. Meanwhile, intramolecular forces like disulfide bonds and salt bridges redistribute energy within subdomains, minimizing overall structural changes.

### Identification of the binding site of CPT drugs on BSA

In order to investigate the binding site for the CPT drugs on BSA, warfarin (as site I marker) and ibuprofen (as site II marker) were used as the site markers in this work. In the competitive experiments of site marker, CPT drugs were gradually added to the co-solution of BSA (1.0 × 10^− 6^ mol/L) and markers (2 × 10^− 6^ mol/L), respectively, and the double-log plots of the CPT drugs quenching effect on BSA fluorescence in the absence and presence of site markers were shown in Fig. S3.

**Table 6:** Binding constant of CPT drugs with BSA in the presence of site markers.

Ligand	Site marker	logK_b_	K_b_ (M ^− 1^)	*R* ^2^
CPT	Blank	3.63 ± 0.48	4265	0.9061
	Ibuprofen	3.12 ± 0.29	1318	0.9618
	Warfarin	2.26 ± 0.36	181	0.9151
10-HCPT	Blank	3.92 ± 1.02	8317	0.8247
	Ibuprofen	4.35 ± 1.18	22,387	0.8579
	Warfarin	2.82 ± 0.49	660	0.9442
TPT	Blank	4.92 ± 0.15	83,176	0.9948
	Ibuprofen	4.90 ± 0.32	79,432	0.9755
	Warfarin	5.18 ± 0.24	151,356	0.9868
CPT-11	Blank	4.81 ± 0.31	64,565	0.9765
	Ibuprofen	4.07 ± 0.31	11,748	0.9650
	Warfarin	3.49 ± 0.39	3090	0.9270

Table 6 summarizes the binding interactions of CPT drugs with BSA in the absence/presence of site-specific markers (warfarin: Site I; ibuprofen: Site II). CPT, 10-HCPT, and CPT-11 show strongest binding in the blank condition (no marker), with affinity K_b_ decreasing significantly in the presence of warfarin (CPT: K_b_ drops from 4265M^− 1^ to 181 M^− 1^; 10-HCPT: K_b_ drops from 8317 M^− 1^ to 660 M^− 1^; CPT-11: K_b_ drops from 64565 M^− 1^ to 3090 M^− 1^), confirming primary binding to Site (I) Moderate competition with ibuprofen suggests partial interaction with Site (II) TPT exhibits atypical behavior, binding affinity increases with warfarin (K_b_ = 151,356 M^− 1^ vs. blank 83,176 M^− 1^) implying potential non-competitive binding or conformational changes enhancing Site I accessibility. 10-HCPT shows an anomalous rise in (K_b_) with ibuprofen (22,387 M^− 1^ vs. blank 8,317 M^− 1^), suggesting experimental interference or indirect effects. High R^2^ values (> 0.9 for most) validate reliable data fitting. These results highlight Site I dominance for most analogs, while TPT’s unique behavior warrants further investigation.

### Molecular docking

BSA is known to have two major drug-binding sites, specifically two hydrophobic cavities located in subdomains IIA and IIIA, known as site I and site II, respectively^56^. For molecular docking simulations using the CHARMM36 force field, the TIP3P water model is generally recommended over SPC due to its explicit parameterization compatibility with CHARMM-based systems. TIP3P better reproduces experimental hydration properties and maintains thermodynamic consistency with CHARMM36^57^.

**Fig. 10:**
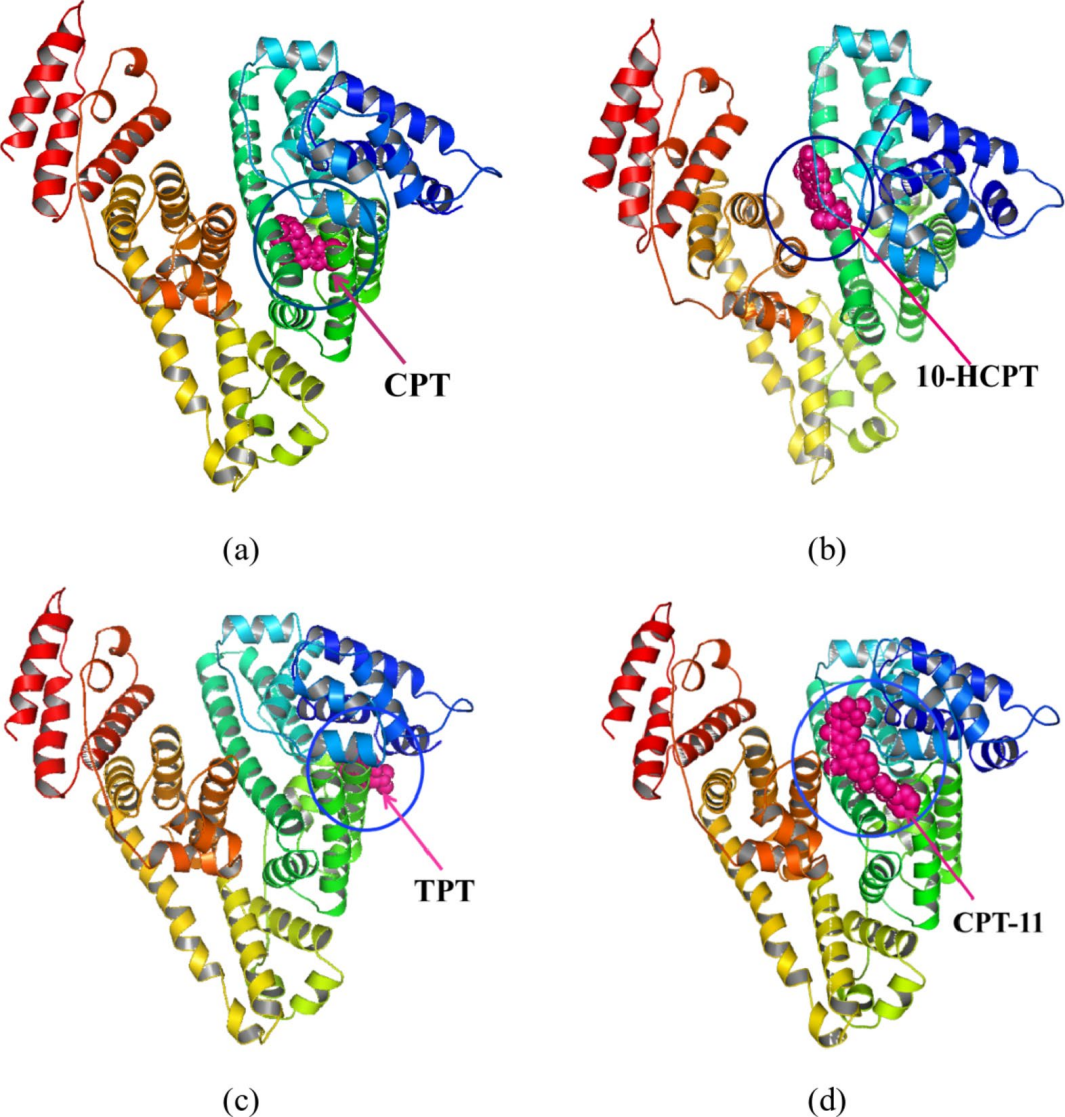
The conformations of (**a**) CPT-BSA, (**b**) 10-HCPT-BSA, (**c**) TPT-BSA, and (**d**) CPT-11-BSA complexes with the lowest energy obtained from molecular docking. BSA is represented by the ribbon structure. CPT, 10-HCPT, TPT, and CPT-11 are represented by sphere model.

To elucidate the preferred binding sites of CPT drugs on BSA and the nature of the interaction forces, molecular docking simulations were conducted using AutoDockTools 1.5.7. The results, shown in Table 7, indicate significant differences in the binding stability of each CPT drug at site I and site II. Generally, CPT drugs exhibit greater binding stability at site I of BSA. The predominant binding configurations of BSA complexes with the four CPT drugs, characterized by the lowest binding free energy ΔG, are illustrated in Fig. 10.

**Table 7:** The energy involved in molecular docking of CPT drugs with BSA.

Ligand	Binding site	ΔE_1_ (kJ·mol^− 1^)	ΔE_2_ (kJ·mol^− 1^)	ΔE_3_ (kJ·mol^− 1^)	ΔG (kJ·mol^− 1^)
CPT	Site I	− 35.33	− 34.79	− 0.54	− 32.86
	Site II	− 31.98	− 31.98	0.0	− 29.47
10-HCPT	Site I	− 37.72	− 37.26	− 0.46	− 32.86
	Site II	− 28.55	− 28.21	− 0.33	− 23.53
TPT	Site I	− 37.38	− 36.58	− 0.79	− 31.14
	Site II	− 30.01	− 25.87	− 1.14	− 23.78
CPT-11	Site I	− 52.87	− 51.86	− 1.01	− 45.38
	Site II	− 38.76	− 33.40	− 5.36	− 31.27

ΔE_1_ is intermolecular interaction energy, which is a sum of van der Waals energy, hydrogen bonding energy, desolvation free energy and electrostatic energy; ΔE_2_ is the sum of van der Waals energy, hydrogen bonding energy and desolvation free energy; ΔE_3_ is electrostatic energy; ΔG is the binding energy change in the binding process.

CPT is an alkaloid with a pentacyclic structure, including a lactone ring and a planar conformation. Its binding energies at Site I and Site II are relatively stable negative values (ΔG = − 32.86 kJ·mol^−1^ and − 29.47 kJ·mol^−1^, respectively), indicating its planar structure fits well within these sites. At Site I, CPT likely engages in favorable π-π stacking with the aromatic rings. The slightly lower stability at Site II may result from spatial constraints less suited to CPT’s elongated conformation. Van der Waals forces and hydrogen bonding also contribute to CPT’s binding stability. 10-HCPT, a 10-hydroxy derivative of CPT, shows slightly more stable binding at Site I (ΔG = − 32.86 kJ·mol^−1^), suggesting the hydroxyl group has minimal impact, possibly due to hydrogen bond formation. However, at Site II, the higher ΔG (− 23.53 kJ·mol^−1^) suggests steric hindrance or less favorable interactions compared to CPT, potentially due to the inability to form beneficial non-covalent interactions. TPT, a semisynthetic CPT derivative with an open side chain, binds relatively stably at Site I (ΔG = − 31.14 kJ·mol^−1^), facilitated by its planar structure allowing π-π stacking and hydrogen bonding. At Site II, the ΔG increases (− 23.78 kJ·mol^−1^), likely due to the open side chain causing unfavorable interactions and steric hindrance, reducing van der Waals forces and other interactions. CPT-11, another CPT derivative with an amide side chain, shows high binding stability at Site I (ΔG = − 45.38 kJ·mol^−1^), likely due to strong interactions facilitated by the amide group, including hydrogen bonding and electrostatic interactions. At Site II, the amide side chain may cause spatial interference, reducing stability (ΔG = − 31.27 kJ·mol^−1^). Despite steric hindrance, CPT-11 maintains greater stability than most other ligands, indicating residual van der Waals and electrostatic interactions contribute to stabilization.

Differences in ligand binding stability at site I or II are primarily influenced by their structural features, spatial compatibility, and interaction types. Larger or strategically positioned substituents may enhance stability by strengthening non-covalent interactions (e.g., hydrogen bonds, electrostatic interactions, van der Waals forces) or reduce it due to steric hindrance or unfavorable interactions.

In this study, we used LigPlot software to simulate the docking of CPT drugs with BSA at the optimal binding site (Site I), creating two-dimensional visualization plots. These plots clearly show interactions such as hydrogen bonds, hydrophobic interactions, and other non-covalent forces between the drug molecules and BSA. Analyzing these visualizations provides insights into the binding modes and interaction forces at Site I, offering important theoretical support for developing BSA-based CPT drug nanoformulations.

**Fig. 11:**
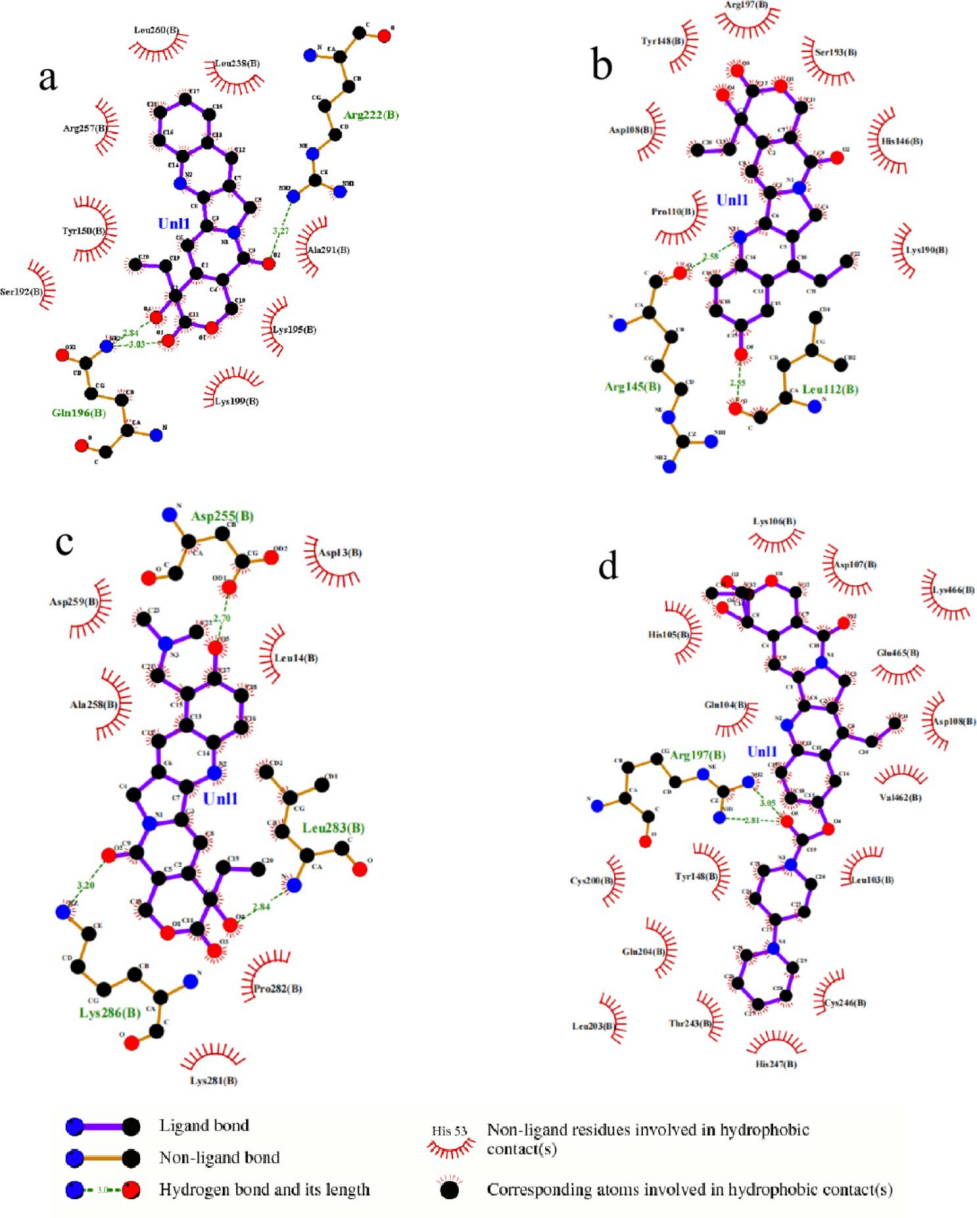
2D visualization of four CPT drugs, (**a**) CPT, (**b**) 10-HCPT, (**c**) TPT and (**d**) CPT−11.

As illustrated in Fig. 11, CPT binds within a hydrophobic pocket in BSA, surrounded by residues Leu-238, Leu-260, Arg-257, Tyr-150, Ser-192, Gln-196, Lys-199, Lys-195, Ala-291, and Arg-222. forming hydrogen bonds with Arg-222 and Gln-196. The 10-HCPT binds in a pocket surrounded by residues Lys-190, His-146, Ser-193, Arg-197, Tyr-148, Asp-108, Pro-110, Arg-145, and Leu-112, forming hydrogen bonds with Arg-145 and Leu-112. TPT binds within a pocket surrounded by Leu-14, Asp-13, Asp-255, Asp-259, Ala-258, Lys-286, Lys-281, Pro-282, and Leu-283, with hydrogen bonds formed at Asp-258, Lys-286, and Leu-283. Lastly, CPT-11 is situated in a pocket involving Leu-103, Val-462, Asp-108, Glu-465, Asp-107, Lys-106, His-105, Gln-104, Arg-197, Tyr-148, Thr-243, His-247, where it forms a hydrogen bond with Arg-197. Overall, the primary forces driving the binding of CPT drugs to BSA are hydrogen bonds and hydrophobic interactions.

The observed discrepancies between experimental and theoretical binding energies stem from fundamental methodological distinctions. Theoretical docking scores reflect gas-phase interactions, whereas experimental ΔG values incorporate solvation and conformational dynamics. For example, CPT-11’s hydrophilic side chain experiences solvent shielding in solution, leading to a less negative experimental ΔG (− 28.56 kJ·mol^−1^) compared to its theoretical value (− 45.38 kJ·mol^−1^). Flexible ligands like 10-HCPT incur additional entropic penalties (ΔΔG ≈ 10.51 kJ·mol^−1^) due to dynamic conformational changes, which rigid docking models neglect. Moreover, super-stoichiometric binding (e.g., TPT: *n* = 1.1165) implies multi-site interactions not resolved in single-pose simulations.

## Conclusion

This study investigates the binding interactions between CPT drugs and BSA using spectroscopic and molecular docking techniques. The results show that CPT drugs form stable complexes with BSA primarily through hydrogen bonding and hydrophobic interactions, inducing conformational changes in the protein. Experimental K_b_ and thermodynamic parameters (ΔG, ΔH, ΔS) reveal distinct interaction mechanisms: TPT binding is driven by hydrogen bonding (ΔH = − 10.96 kJ·mol^− 1^) and hydrophobic interactions, while CPT-11 exhibits stronger binding dominated by electrostatic forces (ΔH = − 86.77 kJ·mol^− 1^) with significant entropy loss (ΔS = − 0.161 kJ·mol^− 1^·K^− 1^). Molecular docking confirms binding at Sudlow site I, aligning with experimental observations. These findings provide valuable insights into the drug-protein interactions of CPT derivatives, supporting the development of albumin-based drug delivery systems with optimized pharmacokinetic properties.

## Materials and methods

### Chemicals and reagents

BSA and topotecan were purchased from Tianjin Baima Technology Co., Ltd.; CPT was obtained from Macklin; 10-hydroxycamptothecin was acquired from Picasso; Irinotecan hydrochloride was purchased from Aladdin. Phosphate buffer solution (pH 7.40) was prepared using sodium dihydrogen phosphate dihydrate (NaH_2_PO_4_·2H_2_O) and disodium hydrogen phosphate dodecahydrate (Na_2_HPO_4_·12H_2_O), both of which were obtained from Tianjin Yongda Chemical Reagent Co., Ltd.2.1 Instrument.

### UV spectroscopy measurements

Absorption spectra were recorded using a UV-vis spectrophotometer (T6 New Century) over the wavelength range of 200–400 nm. The UV spectra of BSA (1.0 × 10^−6^ mol/L) were measured both in the absence and presence of CPT drugs (5.0 ×  10^−6^ mol/L) in an aqueous phosphate buffer solution at 298.15 K.

### Fluorescence measurements

Fluorescence spectra were acquired using a Hitachi F-7100 fluorescence spectrophotometer equipped with a 150 W xenon lamp and 10 mm quartz cuvette. The instrument parameters were maintained at excitation and emission bandwidths of 10 nm, with a scanning speed of 1200 nm·min^− 1^ and PMT voltage of 400 V throughout the experiments. For intrinsic fluorescence measurements, the excitation wavelength was set at 295 nm, and emission spectra were collected in the range of 300–400 nm. The fluorescence spectral changes of BSA (2 × 10^−6^ mol/L) were monitored upon incremental addition of CPT drugs (0–33 × 10^−6^ mol/L, in 3 × 10^−6^ mol/L intervals) in phosphate buffer solution (pH 7.4, 298.15 K). Some data points were excluded for optimal curve fitting.

Three-dimensional fluorescence spectra were acquired for BSA (3 × 10^−6^ mol/L) in both the absence and presence of CPT drugs at molar ratios of 5:1 and 10:1 (CPT drugs: BSA). The excitation wavelength was systematically scanned from 220 to 350 nm with 5 nm increments, while the emission spectra were simultaneously recorded across the 220–500 nm range at a scanning speed of 12,000 nm min^− 1^. The excitation and emission slit widths were maintained at 10 nm throughout the measurements to ensure consistent spectral resolution.

The lifetime fluorescence experiments were performed using a Steady-State/Transient Fluorescence Spectrometer (PL, Edinburgh Instruments FLS980, UK) with the following settings: Excitation wavelength: 295 nm, Emission wavelength: 350 nm. The concentration BSA is 1 × 10^− 6^ mol/L, and CPT drugs concentration is 5 × 10^− 6^ mol/L.

### Molecular docking

The three-dimensional structure of BSA was obtained from the RCSB Protein Data Bank (PDB) database (https://www.rcsb.org/) (PDB ID: 4f5s). The 3D structures of the small molecules CPT (CID: 24360), 10-HCPT (CID: 104842), TPT (CID: 60700), and CPT-11 (CID: 60838) were downloaded from the PubChem database (https://pubchem.ncbi.nlm.nih.gov/). Open Babel 3.1.1 software was utilized to convert the sdf format files from the PubChem database to pdb format. Subsequently, molecular docking simulations were performed to analyze the interaction of CPT drugs with BSA using AutoDockTools 1.5.7 software. Additionally, the molecular visualization was conducted using Pymol version 2.6.0a0 for 3D visualization and LigPlot + version 2.2.8 for 2D visualization.

## Electronic supplementary material

Below is the link to the electronic supplementary material.

Supplementary Material 1

## Data Availability

All data analyzed during the current study are included in this article; further inquiries can be directed to the corresponding authors.
