# Supplementary material for: Spectroscopic and molecular docking studies on binding interactions of camptothecin drugs with bovine serum albumin
Source: Sci Rep. 2025 Mar 7;15:8055. doi: 10.1038/s41598-025-92607-3 (PMC11889159; doi:10.1038/s41598-025-92607-3)
Supplement: Supplementary file 1 — Supplementary Material 1 [file 41598_2025_92607_MOESM1_ESM.docx]

**Supporting materials**

Figure S1

| (a) | (b) | (c) |
| --- | --- | --- |
|   (d) | (e) |  |

Figure S1 Time-resolved fluorescence decay profile of (a) BSA, (b) CPT-BSA, (c)10-HCPT-BSA, (d)TPT-BSA, and (e) CPT-11-BSA. The concentration BSA is 1 × 10^-6^ mol/L, and CPT drugs concentration is 5×10^-6^ mol/L.

Figure S2 .

| 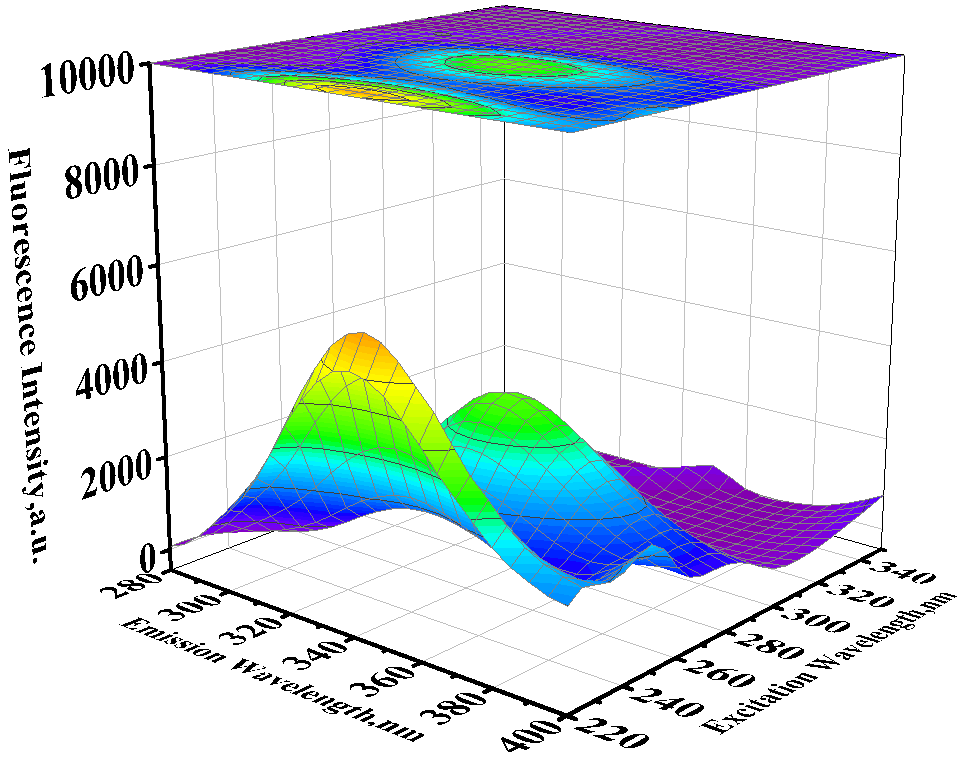  (a) CPT : BSA = 5:1 | 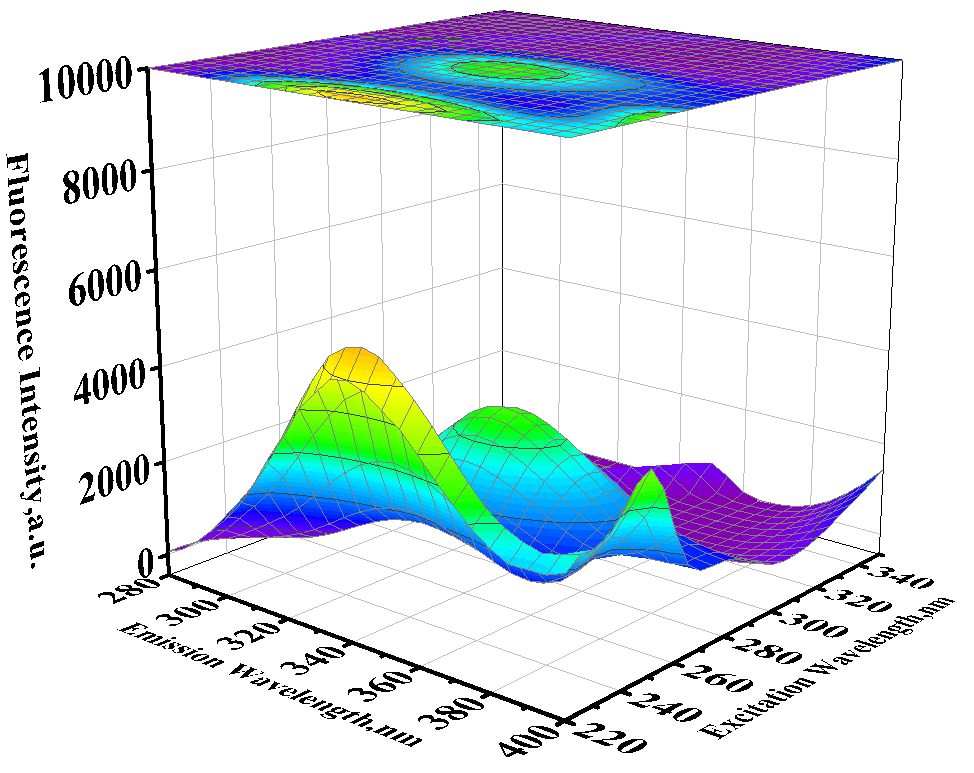  (b) CPT : BSA = 10:1 |
| --- | --- |
| 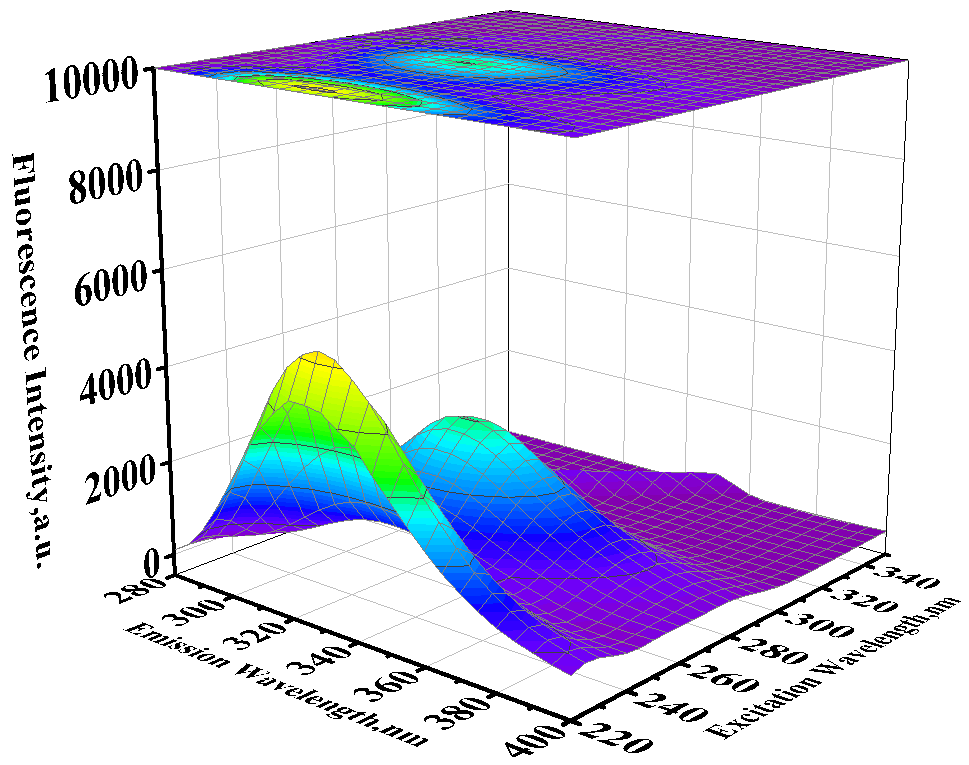  (c) 10-HCPT : BSA = 5:1 | 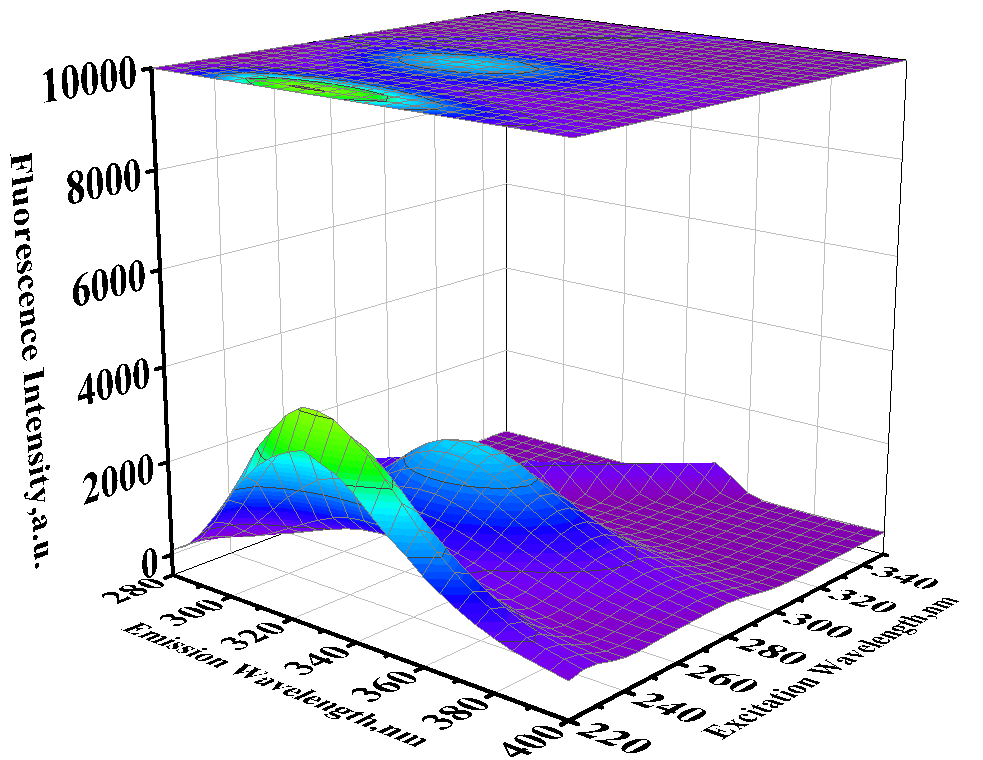  (d) 10-HCPT : BSA = 10:1 |
| 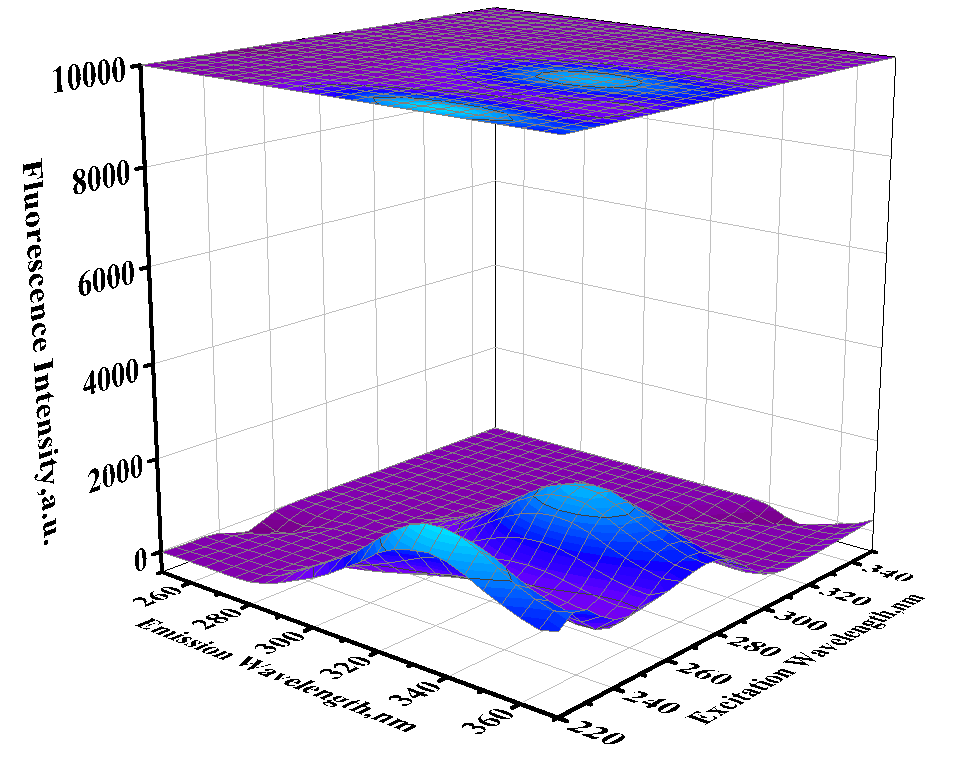  (e) CPT-11 : BSA = 5:1 | 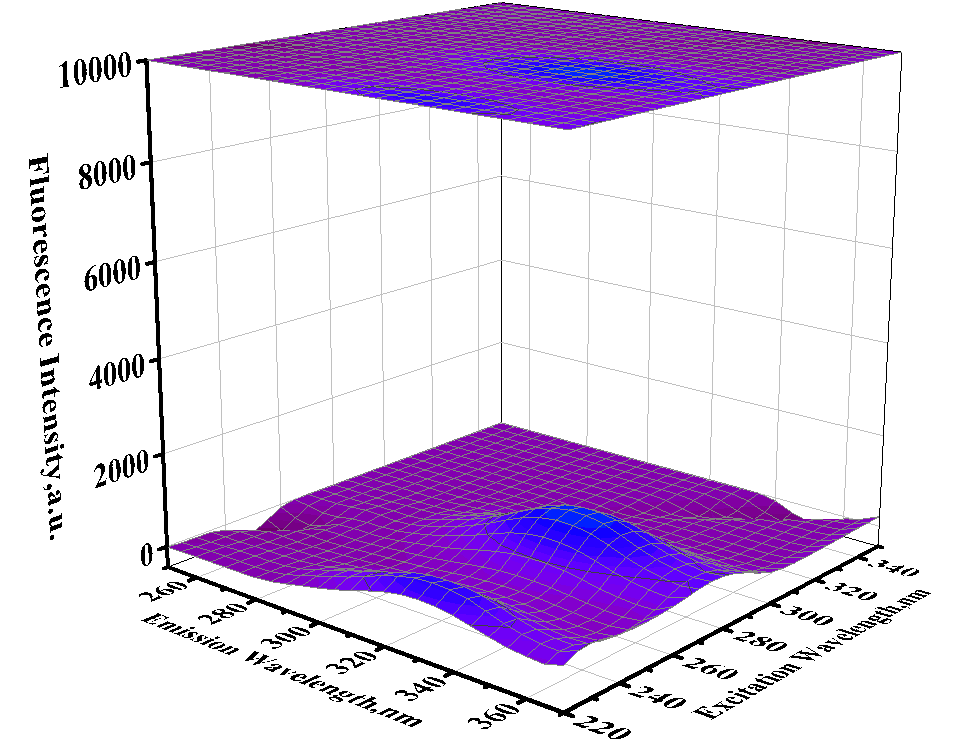  (f) CPT-11 : BSA = 10:1 |

Figure S2 3-D fluorescence spectral projections and corresponding contour maps of (a) CPT : BSA = 5:1, (b) CPT : BSA = 10:1, (c) 10-HCPT : BSA = 5:1, (d) 10-HCPT : BSA = 5:1, (e) CPT-11 : BSA = 5:1 and (f) CPT-11 : BSA = 10:1 systems in aqueous phosphate buffer solution (pH 7.4), concentration of BSA is 3 × 10^-6^ mol/L.

Table S1 3-D fluorescence spectral characteristics of BSA (3 × 10^-6^ mol/L) and CPT drugs–BSA complexes, pH 7.4

| System | drug:BSA | Peak | λ_ex_/λ_em_(nm/nm) | Intensity | Reduction percent | Emission maximum shift |
| --- | --- | --- | --- | --- | --- | --- |
|  | 0 | 1 | 275/340 | 9750 | 0 | 0 |
|  |  | 2 | 225/340 | 7937 | 0 | 0 |
| CPT | 5:1 | 1 | 275/340 | 3382 | 65% | 0 |
|  |  | 2 | 225/340 | 5518 | 30% | 0 |
|  | 10:1 | 1 | 275/340 | 3179 | 67% | 0 |
|  |  | 2 | 225/340 | 5323 | 33% | 0 |
| 10-HCPT | 5:1 | 1 | 275/325 | 2704 | 72% | 15 |
|  |  | 2 | 225/325 | 5002 | 37% | 15 |
|  | 10:1 | 1 | 275/325 | 2170 | 78% | 15 |
|  |  | 2 | 225/325 | 3809 | 52% | 15 |
| TPT | 5:1 | 1 | 275/340 | 8453 | 13% | 0 |
|  |  | 2 | 225/340 | 5380 | 32% | 0 |
|  | 10:1 | 1 | 275/325 | 4956 | 49% | 15 |
|  |  | 2 | 225/320 | 2777 | 65% | 20 |
| CPT-11 | 5:1 | 1 | 275/340 | 2394 | 96% | 0 |
|  |  | 2 | 225/340 | 4370 | 45% | 0 |
|  | 10:1 | 1 | 275/325 | 1937 | 80% | 15 |
|  |  | 2 | 225/325 | 3419 | 57% | 15 |

Figure S3.

|   (a) |   (b) |
| --- | --- |
|   (c) |   (d) |

Figure S3 Double-log plots against log[Q] derived from the quenching of BSA by(a) CPT; (b) 10-HCPT; (c) TPT; and (d) CPT-11, respectively, in aqueous phosphate buffer solution (pH 7.4) in the presence of site markers
